# Expanding the recombinant protein quality in *Lactococcus lactis*

**DOI:** 10.1186/s12934-014-0167-3

**Published:** 2014-12-04

**Authors:** Olivia Cano-Garrido, Fabian L Rueda, Laura Sànchez-García, Luis Ruiz-Ávila, Ramon Bosser, Antonio Villaverde, Elena García-Fruitós

**Affiliations:** Institut de Biotecnologia i de Biomedicina, Universitat Autònoma de Barcelona, Bellaterra, 08193 Cerdanyola del Vallès Spain; Departament de Genètica i de Microbiologia, Universitat Autònoma de Barcelona, Bellaterra 08193 Cerdanyola del Vallès, Spain; CIBER de Bioingeniería, Biomateriales y Nanomedicina (CIBER-BBN), Bellaterra 08193 Cerdanyola del Vallès, Spain; Spherium Biomed S.L., Avda. Joan XXIII, 10, 08950 Esplugues de Llobregat Barcelona, Spain

**Keywords:** *Lactococcus lactis*, Solubility, Recombinant protein quality, Conformational quality, GRAS

## Abstract

**Background:**

*Escherichia coli* has been a main host for the production of recombinant proteins of biomedical interest, but conformational stress responses impose severe bottlenecks that impair the production of soluble, proteolytically stable versions of many protein species. In this context, emerging Generally Recognized As Safe (GRAS) bacterial hosts provide alternatives as cell factories for recombinant protein production, in which limitations associated to the use of Gram-negative microorganisms might result minimized. Among them, Lactic Acid Bacteria and specially *Lactococcus lactis* are Gram-positive GRAS organisms in which recombinant protein solubility is generically higher and downstream facilitated, when compared to *E. coli*. However, deep analyses of recombinant protein quality in this system are still required to completely evaluate its performance and potential for improvement.

**Results:**

We have explored here the conformational quality (through specific fluorescence emission) and solubility of an aggregation-prone GFP variant (VP1GFP) produced in *L. lactis*. In this context, our results show that parameters such as production time, culture conditions and growth temperature have a dramatic impact not only on protein yield, but also on protein solubility and conformational quality, that are particularly favored under fermentative metabolism.

**Conclusions:**

Metabolic regime and cultivation temperature greatly influence solubility and conformational quality of an aggregation-prone protein in *L. lactis*. Specifically, the present study proves that anaerobic growth is the optimal condition for recombinant protein production purposes. Besides, growth temperature plays an important role regulating both protein solubility and conformational quality. Additionally, our results also prove the great versatility for the manipulation of this bacterial system regarding the improvement of functionality, yield and quality of recombinant proteins in this species. These findings not only confirm *L. lactis* as an excellent producer of recombinant proteins but also reveal room for significant improvement by the exploitation of external protein quality modulators.

## Introduction

Obtaining proteins of biotechnological and biomedical interest from their natural sources is hampered by severe economic constraints. The emergence of recombinant DNA technologies allowed developing different gene expression systems (cell factories) adapted to produce functional versions of the desired proteins, becoming recombinant protein production a routine practice in the BioPharma industry [1–3]. Among these expression systems, the bacterium *Escherichia coli* has been the principal working horse, because of its well-known genetics and physiology, cost-effective culture and easy scaling up. However, being *E. coli* a Gram-negative bacterium, cell wall lipopolysaccharides (LPSs) are generic contaminants of the final product and promote not only pyrogenicity but also activation of acute inflammatory responses. The presence of bacterial endotoxins is then one of the major concerns by regulatory agencies [4], and the need of adding steps for endotoxin removal turns otherwise simple processes into practices with high associated costs. Besides, protocols for endotoxin removal can also impair or destroy protein function [5]. Since protein production processes have to meet not only good cost-effectiveness ratios but also high product quality [6–8], the use of cell factories other than *E. coli* is becoming an increasingly recognized need [3]. In this context, other bacterial groups are emerging as intriguing alternatives that offer advantages over the use of *E. coli* regarding protein quality, absence of endotoxins, disulfide bridge formation and solubility [9,10]. Lactic Acid Bacteria (LAB) are classified as Generally Recognized As Safe (GRAS) organisms and represent appealing possibilities for the production of safer therapeutic proteins [11,12]. Specifically, *Lactococcus lactis*, which has been used for long in food industry, has emerged as a cost-effective protein cell factory [12]. In this regard, a wide range of genetic tools adapted to LAB make nowadays possible to successfully produce an increasing number of fully LPS-free recombinant proteins [5,11,13–16]. More specifically, it is worth mentioning that three versatile gene expression systems, named NICE (nisin-controlled expression system) [17], P170 [18,19] and zinc systems [20], have been developed for use in *L. lactis*. Interestingly, *L. lactis* is also currently used as a life vector for drug, DNA and other molecules delivery to mucosal surfaces [15,21–23], proving their huge potential for its use in human medicine [11]. In addition, GRAS organisms are being considered for the production of diverse bacterial products, not only soluble proteins but also biopolymers, polymeric nanoparticles and self-assembling protein-based nanoparticles, among others [24]. For instance, polyhydroxibutirate (PHB) inclusions produced in *L. lactis* are purer than those obtained in *E. coli* [5] and have lower production costs due to the reduction of the number of downstream processing steps [5]. In this context, producing endotoxin-free biopolymeric beads is critical for use in medical applications [25,26].

Despite reports on protein production in *L. lactis* abound [27], further analyses of protein quality and solubility in this system are required to deeply understand the host performance regarding protein quality control and to fully exploit and ever expand, its well-known properties as a factory for soluble, highly functional proteins. Besides, considering that, although *L. lactis* is a facultative anaerobe with a fermentative metabolism, this microorganism is also able to undergo respiratory growth when hemin is added to aerated cultures [28], both growth conditions have also been explored in this study.

## Results

To explore recombinant quality in *L. lactis* we have studied the influence of production time and growth conditions of a wild type strain in the production of an aggregation-prone fluorescent protein (rVP1GFP) that has been previously used as a convenient solubility model in *E. coli* [29]. The specific emission of GFP has been successfully used as a marker of conformational quality of GFP-containing misfolding-prone proteins as there is a positive linear dependence between conformational quality and the presence of native-like conformations [30,31].

### Growth conditions

**Figure 1:**
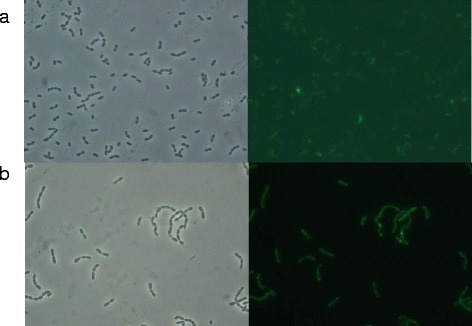
**Optical microscopy images (left) and fluorescence microscopy images (right) of** ***L. lactis*** **NZ9000 overproducing rVP1GFP protein at 30°C 3h post-induction under a) hemin-stimulated respiration and b) anaerobic conditions.** Fluorescent protein aggregates are observed as highly fluorescent dots in the cell cytoplasm.

Under anaerobic fermentation but not under hemin-stimulated respiration, protein solubility was compromised rendering fluorescent protein deposits (Figure 1). This pattern was coincident with that of the formation of PHB inclusions that occurs only under anaerobiosis [26]. In fact, cells were not fluorescent under aerobic conditions (Figure 1), in agreement with previously studies indicating that hemin-induced cell respiration does not support the production of functional proteins [32,33]. Therefore, fermentative growth was established as standard conditions for subsequent experiments.

### Production time

**Figure 2:**
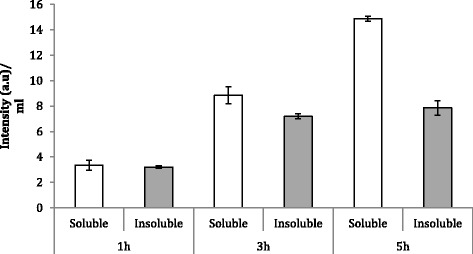
**Fluorescence at 1, 3 and 5 h post-induction of soluble rVP1GFP (white bars) and insoluble rVP1GFP (grey bars) produced in** ***L. lactis*** **NZ9000 at 30°C.**

**Figure 3:**
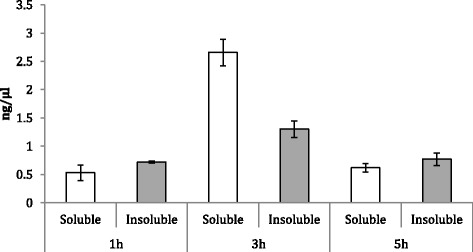
**Yield of soluble (white bars) and IB rVP1GFP (grey bars) produced in** ***L. lactis*** **NZ9000 at 30°C.**

**Figure 4:**
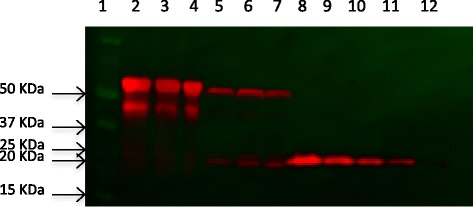
**Western blot of soluble and insoluble rVP1GFP fractions produced in** ***L. lactis*** **NZ9000.** 1: Marker, 2: insoluble rVP1GFP (replica 1), 3: insoluble rVP1GFP (replica 2), 4: insoluble rVP1GFP (replica 3), 5: soluble rVP1GFP (replica 1), 6: soluble rVP1GFP (replica 2), 7: soluble rVP1GFP (replica 3), 8-12: standard curve (750, 500, 250, 125 and 75 ng of R9-GFP). The band around 50 Kda corresponds to VP1GFP , while the band observed around 20 KDa corresponds to GFP.

**Figure 5:**
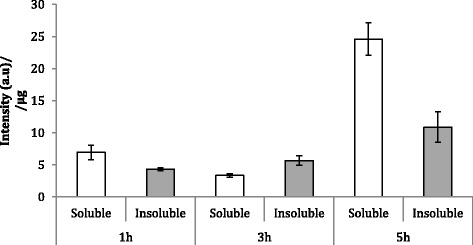
**Specific fluorescence of soluble (white bars) and insoluble (grey bars) rVP1GFP produced in** ***L. lactis*** **NZ9000 at 30°C.**

The fluorescence emission (Figure 2) and the ratio of soluble versus insoluble protein (Figure 3) fractions were determined by fluorimetry and western blot (Figure 4), respectively. The fluorescence emission does not present significant differences at different production times. However, in general terms, it is possible to conclude that GFP activity increases at longer production times, being this effect more marked in the soluble fraction (Figure 2). Besides, it is worh mentioning that at 3 h post-induction, protein solubility reached 67%, a yield much higher than that reached in *E. coli* for the same protein under comparable conditions (10-18%, [34]). In this context, the potential of the system for the soluble protein production was confirmed with a difficult-to-express human catalase, which under these conditions majorly occurred at the soluble cell fraction (75%) (data not shown). On the other hand, the conformational quality, namely the ratio between protein activity and protein yield, of soluble rVP1GFP (estimated through its specific fluorescence) (Figure 5) evolved contrarily to the resulting protein amount in cells (Figure 2). This means that those conditions that favour protein solubility are those giving non-optimal results regarding conformational quality. Interestingly, such a divergence between protein yield and quality has been previously described in recombinant *E. coli* [29,35], but the present finding confirms this fact as a generic event.

### Growth temperature

Finally, we determined how the production at suboptimal temperatures might influence solubility and conformational quality, through fluorescence determination (Table 1). The fluorescence of the soluble and insoluble protein produced at 25°C was similar to that obtained at 30°C. However, the specific fluorescence at 30°C was notably higher than that observed at 25°C, showing in this last condition a 6-fold increase and 1.6-fold increase for the soluble and insoluble fraction respectively. At 16°C we observed a decrease in the fluorescence of both soluble and insoluble protein fractions. On the other hand, while the specific activity of the insoluble protein at 16°C was practically the same than that obtained at 30°C, the specific fluorescence of the soluble fraction at 16°C improved. Thus, decreasing the production temperature did not result in any improvement of fluorescence emission of insoluble protein, which was highly fluorescent at 30°C. In this direction, we also observed residual enzymatic activity in the insoluble fraction of catalase-producing cells (6.5 μmol/min/μg) (data not shown).

**Table 1:** **Effect of growth temperature on protein solubility and conformational quality**

		**Fluorescence (A.U./ml · OD) %**	**Specific fluorescence (A.U./μg) %**
**30°C**	**Soluble fraction**	18,32	59,40
	**Insoluble fraction**	81,68	40,60
**25°C**	**Soluble fraction**	15,19	26,26
	**Insoluble fraction**	84,81	73,74
**16°C**	**Soluble fraction**	0,02	68,09
	**Insoluble fraction**	99,98	31,91

## Discussion

Under the need to obtain LPS-free bacterial-derived products for biomedical applications, the use of Gram-positive microorganisms as a recombinant cell factory has progressively gained relevance [12]. In this context, an increasing number of approaches using LAB and other Gram-positive microorganisms [9], have proven the huge potential of these cell factories in recombinant protein production [12,36–38]. Although *L. lactis* has been widely explored as a cell platform for the production of both homologous and heterologous recombinant proteins [39–41], as far as we know, the solubility and fine conformational quality of both soluble and insoluble recombinant protein has been never studied in detail in this microorganism.

In the present study, we provide data in line with previous studies that describe that fermentative growth is the optimal condition to successfully produce recombinant proteins in *L. lactis* (Figure 1) [32,33]. Thus, although hemin-stimulated respiration has been described as an alternative growth condition inducing an increase in biomass and reducing media acidification [42], our results clearly show that under this condition protein production is highly compromised in both soluble and insoluble protein fractions. These data are in agreement with previous results published. Specifically, the authors describe the reduction of recombinant protein production under aerobic conditions in the presence of hemin, when compared to that obtained under similar conditions without aeration [32,33].

Growth temperature also plays a key role regulating protein solubility and conformational quality. In this regard, protein activity is negatively affected at low temperatures, but the conformational quality of soluble protein reaches higher values at suboptimal temperatures, especially at 16°C. On the contrary, specific activity of insoluble protein is poorly influenced by the temperature.

## Conclusions

Recombinant protein quality in *Lactococcus lactis* is largely influenced by the metabolic regime, growth conditions and temperature. By controlling these parameters, the conformational quality of both soluble and insoluble protein fractions is widely modulated, what offers a desirable high versatility of this system for the production of endotoxin-free conventional soluble protein and protein aggregates of biomedical interest. Beyond the well-recognized ability of this system as a producer of soluble proteins, the wide range of conformational quality of the model protein prompts the further exploitation of external effectors as efficient solubility modulators for optimal yields.

## Materials and methods

### Bacterial strain, plasmids and growth conditions

The *Lactococcus lactis* strains used in this study was NZ9000 (*pepN::nisRnisK*) (NIZO). A model recombinant protein rVP1GFP (a fusion between the VP1 capsid protein from the food-and-mouth disease virus and the Green Fluorescent Protein) and human catalase (hcatalase) were produced by expressing the encoding gene from the Cm^R^ pNZ8148 plasmid (NIZO) under *nis*A promoter control.

### Plasmid construction

The rVP1GFP and hcatalase sequences were codon optimized (*Geneart*). In the sequence design we added a *NcoI* restriction site at 5’ followed by nucleotides CA to restore the reading frame and an *PstI* restriction site at 3’ for rVP1GFP and *XbaI* for hcatalase. rVP1GFP gene was digested by *Nco*I and *PstI*, and ligated into the *NcoI-PstI* fragment of the expression plasmid pNZ8148. hcatalase gene was digested by *Nco*I and *XbaI*, and ligated into the *NcoI-XbaI* fragment of the expression plasmid pNZ8148. Ligation products were transformed by electroporation into *L. lactis* competent cells and positive colonies were isolated by antibiotic selection.

### Preparation of *L. lactis* competent cells

A protocol to prepare competent *L. lactis* was developed based on previous data [43]. *L. lactis* was growth O/N at 30°C in 50 ml M17 broth media enriched with 0.5% glucose, 2% glycine, 0.5 M sucrose (G-SGM17B) and appropriated antibiotics. Then, 400 ml of G-SGM17B medium were inoculated with an aliquot of the overnight culture to reach an OD_550 nm_ = 0.05 and they were grown at 30°C during around 3h until the OD_550 nm_ reached 0.2-0.3. Then, cells were harvested by centrifugation at 10,000 g for 20 min at 4°C and the pellet was resuspended in 400 ml of 0.5 M sucrose plus 10% glycerol. Samples were harvested again at 10,000 g for 10 min at 4°C, and the pellet was washed in 200 ml of 0.5 M sucrose plus 10% glycerol and 50 mM EDTA and formed again at 10,000 g for 10 min and 4°C. Cells were resuspended in 100 ml of 0.5 M sucrose plus 10% glycerol, and pelleted by last time at 10,000 g for 10 min at 4°C. Finally, the sediment was suspended in 4 ml of 0.5 M sucrose plus 10% glycerol, aliquoted and stored at -80°C until used in transformation experiments.

### Transformation

Electroporation was performed using Gene Pulser from Bio-rad fitted with 2500V, 200 Ω and 25 μF in a pre-cooled 2 cm electroporation cuvette. Following, samples were supplemented with 900 μl restorative medium (M17 broth with 0.5% Glucose, 20 mM Mg_2_Cl_2_ and 2 mM Ca_2_Cl_2_) and incubated for 2h at 30°C. The electroporation mix was centrifuged for 10 min at 10,000 g at 4°C and the pellet was resuspended in 100-200 μl of M17 media and plated.

### Recombinant protein production

A pre-inoculum was prepared from a fresh colony of the recombinant cells the pre-inoculum was used to inoculate 250 ml shake flasks. The cells were cultured as described in [44]. Specifically, chloramphenicol (5 μg/ml) was used for plasmid maintenance and an agitation of 250 rpm was used for those cultures grown under hemin-stimulated respiration.

Expression of the target gene was induced when the optical density reached a value of 0.5 with nisin, as described in [17,44]. Growth temperatures in the production phase were 30, 25 or 16°C. Samples were taken at 1, 3 and 5 h in cultures at 30°C and at 5 h post-induction in cultures grown at 25°C and 16°C.

All the experiments have been run in triplicate (replica 1, replica 2 and replica 3).

### Protein fractioning

Samples of 10 ml were taken in triplicate from bacteria cultures. Cells were pelleted by centrifugation at 10,000 g at 4°C for 10 min and the sediment was resuspended in 1 ml phosphate buffered saline (PBS) supplemented with (Complete EDTA-free, Roche) to prevent proteolysis. Then, ice-jacketed samples were disrupted by sonication (3 cycles of 5 min at 40% amplitude under 0.5 s cycles). Total cell extracts were centrifuged at 15,000 g and 4°C for 15 min. Finally, soluble and insoluble fractions were aliquotated until further analysis.

### Protein determination

Soluble and insoluble protein fractions were analysed by denaturing SDS-PAGE (10% acrylamide). Samples were resuspended with denaturing buffer (Laemli 4x: Tris base 1.28 g, glycerol 8 ml, SDS 1.6 g, β-mercaptoethanol 4 ml, urea 9.6 g in 100 ml) [45]. Soluble and insoluble protein fractions were boiled for 5 and 45 min respectively. At that time, samples were loaded onto the gel. SDS-PAGE protein bands were electroblotted onto nitrocellulose membranes and identified using a commercial polyclonal serum against GFP (GFP (FL): SC-833, Santa Cruz Biotechnology). The secondary antibody was an anti‐rabbit (Goat Anti-Rabbit IgG (H + L)-HRP Conjugate #172-1019, Bio Rad). The amounts of recombinant protein were estimated by comparison with known amounts (usually ranging from 75 to 750 ng) of R9-GFP [46] or commercial hcatalase (Sigma Ref. C9322). Densitometric analyses of the bands were performed with the Quantity One software.

### Fluorimetry

Fluorescence emission (510 nm) was determined by fluorimetry using a Cary Eclipse Fluorescence Spectrophotometer (Variant), by using an excitation wavelength of 450 nm, to measure protein fluorescence at soluble and insoluble protein.

### Catalase activity assay

Catalase activity was determined by the Catalase Assay kit (Abcam Ref. ab83464). In the assay H_2_O_2_ was added to the catalase samples in order to measure the unconverted H_2_O_2_ which reacts with OxiRed™. Reaction final product was measured at Ex/Em = 535/587 nm by Victor 3 Multilabel Plates Reader. Catalase activity is reversely proportional to the signal.

### Microscopy

Samples of 1 ml were taken from bacteria cell culture by triplicate and harvested by centrifugation at 10,000 g and 4°C for 10 min. Cells were fixed with 0.1% formaldehyde in PBS and kept at 4°C until microscopy observation. Fixed cells were deposited on a glass slide fixed with a slide cover and observed with a Leica DRMB Microscopy. Microphotographies were taken by phase contrast and epifluorescense.
